# The N-Terminal Domain of Spike Protein Is Not the Enteric Tropism Determinant for Transmissible Gastroenteritis Virus in Piglets

**DOI:** 10.3390/v11040313

**Published:** 2019-03-30

**Authors:** Gang Wang, Rui Liang, Ziwei Liu, Zhou Shen, Jiale Shi, Yuejun Shi, Feng Deng, Shaobo Xiao, Zhen F. Fu, Guiqing Peng

**Affiliations:** 1 State Key Laboratory of Agricultural Microbiology, College of Veterinary Medicine, Huazhong Agricultural University, Wuhan 430070, China; alvin_ssd@webmail.hzau.edu.cn (G.W.); liangrui0123@webmail.hzau.edu.cn (R.L.); lzw.hzau.edu.cn@webmail.hzau.edu.cn (Z.L.); 15527034148@163.com (Z.S.); sjl@webmail.hzau.edu.cn (J.S.); shiyuejun2017@mail.hzau.edu.cn (Y.S.); dengfeng207@163.com (F.D.); vet@mail.hzau.edu.cn (S.X.); zhenfu@uga.edu (Z.F.F.); 2 Key Laboratory of Preventive Veterinary Medicine in Hubei Province, The Cooperative Innovation Center for Sustainable Pig Production, Wuhan 430070, China; 3 Departments of Pathology, College of Veterinary Medicine, University of Georgia, Athens, GA 30602, USA; 4 College of Life Science and Technology, Huazhong Agricultural University, Wuhan 430070, China

**Keywords:** transmissible gastroenteritis virus, spike gene, enteric tropism, reverse genetics, CRISPR/Cas9

## Abstract

Transmissible gastroenteritis virus (TGEV) is the etiologic agent of transmissible gastroenteritis in pigs, and the N-terminal domain of TGEV spike protein is generally recognized as both the virulence determinant and enteric tropism determinant. Here, we assembled a full-length infectious cDNA clone of TGEV in a bacterial artificial chromosome. Using a novel approach, the clustered regularly interspaced short palindromic repeat (CRISPR)/CRISPR-associated protein 9 (Cas9) systems efficiently and rapidly rescued another recombinant virus with a 224-amino-acid deletion in the N-terminal domain of the TGEV *Spike* gene (S_NTD224), which is analogous to the N-terminal domain of porcine respiratory coronavirus. S_NTD224 notably affected the TGEV growth kinetics in PK-15 cells but was not essential for recombinant virus survival. In animal experiments with 13 two-day-old piglets, the TGEV recombinant viruses with/without S_NTD224 deletion induced obvious clinical signs and mortality. Together, our results directly demonstrated that S_NTD224 of TGEV mildly influenced TGEV virulence but was not the enteric tropism determinant and provide new insights for the development of a new attenuated vaccine against TGEV. Importantly, the optimized reverse genetics platform used in this study will simplify the construction of mutant infectious clones and help accelerate progress in coronavirus research.

## 1. Introduction

Coronaviruses (CoVs) are single-stranded, positive-sense RNA viruses closely related to animal and human health [1,2,3]. CoVs belong to the *Coronaviridae* family, which consists of the *Alpha-, Beta-, Gamma-*and *Deltacoronavirus* genera [4]. Since 2003, CoVs, including severe acute respiratory syndrome CoV (SARS-CoV), Middle East respiratory syndrome CoV (MERS-CoV), and porcine epidemic diarrhea virus (PEDV), have swept across the world and caused considerable global economic losses [5,6,7,8,9]. As the largest RNA genome viruses, CoVs have at least six typical overlapping open reading frames (ORFs), which encode polyprotein 1a (pp1a), polyprotein 1ab (pp1ab), spike (S) glycoprotein, envelope (E) protein, matrix (M) protein, and nucleoprotein (N) [10,11].

Transmissible gastroenteritis virus (TGEV), one of the representative CoVs of the *Alphacoronavirus* genus, is the etiologic agent of transmissible gastroenteritis (TGE) in pigs [12]. TGEV is widespread in the pork industry, causes high mortality in neonatal pigs, and is generally thought to share a common ancestor with porcine respiratory coronavirus (PRCV) [13]. Both TGEV and PRCV have a common cell receptor, aminopeptidase N (APN), but a coreceptor (Neu5Gc) of TGEV is recognized to confer enteric tropism to TGEV [14,15,16,17]. In addition, TGEV always causes severe diarrhea, whereas PRCV usually causes mild or no clinical signs [18]. Compared with TGEV *Spike* gene, large deletions (200–230 aa) were found in the N-terminal domain of PRCV *Spike* gene [19]. The N-terminal domain of the S-glycoprotein is considered to be responsible for the different clinical signs of TGEV and PRCV and is generally recognized as not only the virulence determinant but also an enteric tropism determinant [13,20]. Previous research has shown that PRCVs are likely derived from an N-terminal amino-acid deletion of the TGEV Spike protein [15,21], but direct evidence obtained from reverse genetics is still needed to confirm this hypothesis.

Since the first successful construction of TGEV infectious clones, the various reverse genetic systems that have been developed have made a considerable contribution to CoV research, particularly research in MERS, SARS, TGEV, and PEDV [22,23,24,25,26,27]. Among the various reverse genetics methods for CoVs, transfection of the full-length infectious cDNA clone and cotransfection of the full-length RNA and N gene transcripts are the most commonly adopted methods for the rescue of recombinant viruses [25,26,28,29,30]. However, these methods usually require the manipulation of several cDNA fragments or plasmids for the construction of a new mutant CoV [31]. Although targeted RNA recombination or the Red-mediated recombination strategy might partly accelerate or simplify the process of constructing an infectious recombinant virus, the selection of the correct positive clone is also a time-consuming process [32,33,34,35]. In fact, the limitations of the various currently available traditional reverse genetics methods for CoV genome manipulation, such as ligation with several cDNA fragments in vitro, targeted RNA recombination, division of toxic gene sequence propagation in bacteria, or recombinant vaccinia virus vectors, have severely hampered the speed and efficiency of developing CoV reverse genetics techniques [23,36,37,38].

CRISPR/Cas systems, which constitute a recent new gene editing technology developed from the RNA-mediated adaptive defense systems evolved by bacteria and archaea, have been applied to a wide variety of organisms for the in vivo editing of large genomes [39,40,41]. Furthermore, some studies have investigated the in vitro editing of DNA fragments or plasmids [42,43,44]. In general, the CRISPR/Cas system from *Streptococcus pyogenes*, namely, the SpyCas9 protein with a mature single-guide RNA (sgRNA), is used for in vitro DNA cleavage [42]. However, the in vitro editing of RNA virus genomes, particularly viruses with large RNA genomes, such as CoVs, using this method has not been reported.

In this study, we successfully constructed a TGEV infectious clone and utilized the molecular scissors of the CRISPR/Cas9 system to rescue another TGEV mutant with S_NTD224 deletion [45]. Through animal experiments involving challenge with two types of rescued recombinant TGEV viruses, we elucidated that TGEV S_NTD224 was not the determinant in viral enteric tropism and pathogenesis.

## 2. Materials and Methods

### 2.1. Cells and Viruses

PK-15 cells were cultured in Dulbecco’s modified Eagle’s medium (Gibco, Waltham, MA, USA) supplemented with 10% fetal bovine serum at 37 °C with 5% CO_2_. TGEV strain WH-1 (GenBank accession number HQ462571) was propagated at 37 °C in a 5% CO_2_ incubator in Dulbecco’s modified Eagle’s medium (Gibco, Waltham, MA, USA) supplemented with 2% fetal bovine serum (Gibco, Waltham, MA, USA). All the experiments using live viruses were performed under biosafety level (BSL) 2 conditions.

### 2.2. Amplification of TGEV cDNAs and Sequence Analysis

Total RNA was extracted from virus-infected cultures using TRIzol reagent (Invitrogen, Carlsbad, CA, USA), and cDNA was reverse transcribed with reverse transcriptase (Takara, AMV, Kusatsu, Japan) using random primers (Takara, Kusatsu, Japan, 6 mer). All the fragments were amplified by polymerase chain reaction (PCR) with Phanta Super Fidelity DNA polymerase (Vazyme, Nanjing, Jiangsu, China).

The natural complete genome of TGEV WH-1 was determined by sequencing (GenScript, Nanjing, China) the overlapping PCR products cloned into the corresponding vectors in triplicate. Compared with the parental TGEV WH-1 from NCBI (Bethesda, MD, USA), several site mutations, including T6299C, G11123T, G25943C, C26094T, and C26336G, were observed, and these mutations were maintained during the cloning of the TGEV full-length genome. A point mutation, A4553T, was introduced by overlap extension PCR to remove the natural Van91I site and maintained as the rescue marker. As described in previous research, the *EGFP* gene was inserted into the TGEV genome [46,47] to replace the original sequence positioned from 24,826 to 28,580.

### 2.3. Construction of the TGEV Subclones

The virus genome was divided into six continuous fragments (A to F), and each fragment was amplified from the total cDNA using specific primers (available upon request). Fragments A, B, D, and E were cloned into the pMD18-T vector (Takara, Kusatsu, Japan). Fragment A was cloned with the SacI and Van91I sites, and fragment E was cloned with the Van91I and KasI sites. Notably, fragment A was cloned to contain a SacI site (***GAGCTC***GTTTAGTGAACCGT) [48] located in the 5′ terminal of the TGEV genome sequence. Fragments B and D were cloned with Van91I sites. Fragment C was cloned into a BAC plasmid (kindly provided by Prof. Cao Gang) that was modified from pBeloBAC11 to include a Van91 site [36]. Fragment F was also cloned into the BAC plasmid with SacI and KasI sites introduced at the 5′ and 3′ termini of the TGEV genome, respectively. As the final recipient BAC vector, subclone F also contained the synthesized essential element sequences, such as the CMV promoter, the poly(A) tail sequence (25A), the HDV RZ sequence (hepatitis delta virus self-cleaving ribozyme sequence, Rz), and the bovine growth hormone (BGH) transcription terminal signal (GenScript, Nanjing, China) [49].

### 2.4. Assembly of Full-length TGEV Infectious Clone

After the six subclones were sequenced in their corresponding vectors, subclones A and E were first digested with SacI and KasI, respectively, and then treated with calf intestinal alkaline phosphatase (CIAP, Scientific). All the subclones were then digested with Van91I except subclone F, which was digested with SacI and KasI. Subsequently, all the digested products were purified with a gel extraction kit (Omega, Norcross, GA, USA), and fragments A to F were ligated for more than 2 h at 16 °C and transformed into chemically competent DH10B cells (Biomed, Beijing, China). After determination of all the fragments by bacterial PCR, the positive clones were further determined by restriction fragment length polymorphism with KpnI, and the correct clone was designated pTGEV-GFP BAC after sequencing (GenScript, Nanjing, China).

### 2.5. Rescue of the TGEV-GFP Infectious Clone in PK-15 Cells

PK-15 cells were seeded in a six-well plate and incubated for 12 h, and the recovery of TGEV-GFP or TGEV-GFP-ΔS_NTD was then performed by transfecting 5 µg of the corresponding BAC into PK-15 cells with 8 µL of Lipofectamine 2000 (Invitrogen, Carlsbad, CA, USA). At 48 h post-transfection, the collected virus progenies were purified once by fluorescent plaques. Subsequently, the purified virus clone was amplified and stored until use at −80 °C.

### 2.6. sgRNA Generation and Evaluation of Its Transcript Integrity and Quantity

For the design of a sgRNA to mediate cleavage of the targeted site, a constant reverse primer (ssDNA-R) and two forward primers (ssDNAa-F and ssDNAb-F) specific for sites a and b were synthesized as shown in Table 1, similar to the protocol described in a previous report [45]. To anneal the primers ssDNAa-F and ssDNAb-F with the reverse primer ssDNA-R, PCR was conducted for 30 cycles at 95 °C for 15 s, 55 °C for 15 s, and 72 °C for 20 s using 2× UTaq MasterMix (Zoman). The PCR products were then purified with CP buffer (Omega, Norcross, GA, USA) and transcribed using a T7 transcription kit (NEB, Ipswich, MA, USA) according to the manufacturer’s instructions to produce the targeted sgRNA a and sgRNA b. The purity of the sgRNA products was analyzed by electrophoresis on agarose gels using 0.5 μg of each sgRNA product.

### 2.7. Specific Cleavage of pTGEV-GFP BAC by the CRISPR/Cas9 System In Vitro

To modify the sequence of TGEV S_NTD224, pTGEV-GFP BAC was digested using the targeted sgRNAs. Specifically, pTGEV-GFP BAC was digested in a 50 µL reaction mixture with 5 µg of pTGEV-GFP BAC, 5 µL of Cas9 (NEB, Ipswich, MA, USA), 10 µg of sgRNA, and 5 µL of nuclease reaction buffer incubated at 37 °C for more than 2 h or, preferably, overnight.

### 2.8. Construction and Recovery of the Recombinant Virus Containing the S_NTD224 Mutation

For purification of the digested pTGEV-GFP BAC, an equivalent volume of Solution I (plus RNase; Omega, Norcross, GA, USA) was first added to digest the sgRNA at room temperature for 3 min, and the CP buffer (OMEGA, Norcross, GA, USA) was then applied to recycle the digested BAC according to the manufacturer’s instructions. The PCR products with the 672-bp deletion were constructed by two-cycle PCR. First, the primers rec-672SF and δS-NTDR or the primers rec-672SR and δS-NTDF (Table 2) were used to amplify the primary PCR products from the template of the pTGEV-GFP BAC. Second, the two primary PCR products were annealed to produce PCR products of the 672-bp deletion using the primers rec-672SF and rec-672SR (Table 2). Homologous recombination was then performed using the ClonExpress II One Step Cloning Kit (Vazyme, Nanjing, Jiangsu, China) according to the manufacturer’s instructions using 200 ng of recycled linearized pTGEV-GFP BAC, 45 ng of PCR products of S_NTD and two pairs of primers (rec-672SF/δS-NTDR and rec-672SR/δS-NTDF) (Table 2). Subsequently, a pair of primers (PrimerF/PrimerR) (Table 2) was designed to amplify the sequence of the modified S_NTD224 area for sequencing (GenScript, Nanjing, China). The recombinant virus corresponding to the correct pTGEV-GFP-ΔS_NTD BAC was then recovered as described above.

### 2.9. Growth Curves of Viruses

PK-15 cells were infected with TGEV WH-1 or a recombinant virus (TGEV-GFP or TGEV-GFP_ΔS_NTD) at a multiplicity of infection (MOI) of 0.01 in six-well plates for 1 h and then washed three times with phosphate-buffered saline (PBS). Subsequently, the supernatants of the infected cells at 12, 24, 36, and 48 h post-infection were collected and stored at −80 °C. The viral titers at each time point were determined by TCID_50_. The viral titers of the two recombinant viruses could also be determined by obvious green fluorescence at 24 h post-infection, but a more obvious CPE of the virus could be observed at 48 h post-infection.

### 2.10. Viral Fluorescent Plaque Assay

PK-15 cells in six-well plates were inoculated with 10-fold serially diluted recombinant virus. After virus adsorption for 30 min, monolayer cells were washed three times with PBS and overlaid with a mixture of 2% low-melt agarose and 2 times the concentration of DMEM (Invitrogen, Carlsbad, CA, USA) supplemented with 4% fetal bovine serum (Gibco). The overlay was then solidified at 4 °C for 10 min. Subsequently, the plates were cultured at 37 °C in a 5% CO_2_ incubator, and 4 days post-infection, the fluorescent plaques were visualized by fluorescence microscopy.

### 2.11. Animal Experiments with Piglets

Thirteen 2-day-old piglets from a TGEV-free sow were randomly divided into three groups and fed fresh liquid milk diluted in warm water every 4 h. All piglets were confirmed to be free of TGEV, PEDV, porcine delta coronavirus (PDCoV), and rotavirus (RV) through a RT-PCR assay of piglet feces before viral challenge. The piglet weights were measured and recorded at the beginning of the challenge. The piglet challenge group was intranasally and orally inoculated with 500 μL (1 × 10^5^ TCID_50_) of chimeric virus, and the mock-infected control group was intranasally and orally inoculated with 500 μL of DMEM.

The piglets were monitored for their clinical status every 4 h. Any piglet exhibiting moribund signs were euthanized. At 7 days post-inoculation, all surviving piglets were euthanized consecutively to reduce the stress of the other piglets. Before necropsy, the weight of each piglet was recorded. At necropsy, five sections of the duodenum, jejunum, ileum, colon and stomach were collected, fixed in 10% formalin for histopathological examination and stained with hematoxylin and eosin (HE). After necropsy, samples of jejunal contents and lung tissue were collected for virus detection by nested RT-PCR using the specific primers F1/R1 and F2/R2 (Table 2) [50].

### 2.12. Ethics Statement

The animal experiments were performed according to the protocols approved by The Scientific Ethics Committee of Huazhong Agricultural University (Permit number: HZAUSW-2017-007). The animal care and maintenance protocols complied with the recommendations detailed in the Regulations for the Administration of Affairs Concerning Experimental Animals made by the Ministry of Science and Technology of China.

## 3. Results

### 3.1. Design of a TGEV Infectious Clone and Rescue of the Recombinant Virus

**Figure 1:**
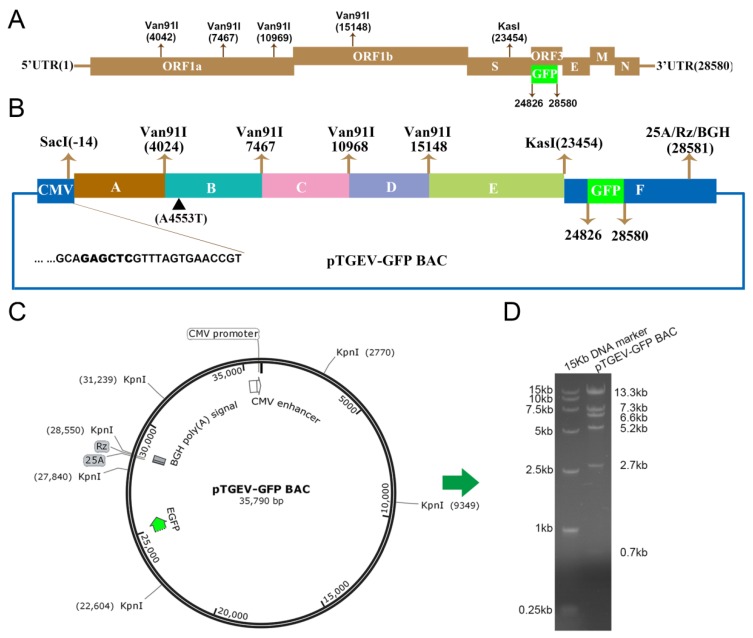
Construction of the TGEV-GFP infectious bacterial artificial chromosome (BAC) clone. The number represents the nucleotide (nt) position in the TGEV genome. (**A**) Structure of the TGEV genome. The 5′ and 3′ UTRs represent the 5′ and 3′ untranslated regions, respectively. (**B**) The TGEV genome was divided into six contiguous cDNAs (A to F): A, −14 to 4024; B, 4025 to 7467; C, 7468 to 10,968; D, 10,969 to 15,148; E, 15,149 to 23,454; and F, 23,455 to 28,580. A4553T was introduced to ablate a natural Van91I site at nt 4553 (▲). The CMV promoter, fragment F, a 25-bp poly(A) tail (25A), hepatitis delta virus self-cleaving ribozyme sequence, Rz (HDV RZ), and the bovine growth hormone (BGH) transcriptional terminal signal were inserted into the BAC to form the final recipient BAC vector or subclone F. The following restriction sites are noted: SacI (−14), Van91I (4024, 7467, 10,968, and 15,148) and KasI (23,454). The *EGFP* gene replacing open reading frame 3 (*ORF3*) is noted at 24,826 and 25,692. (**C**) Schematic map of pTGEV-GFP BAC restriction enzyme digestion by KpnI. (**D**) The left lane is the DL15000 DNA marker, and the right lane is the product of pTGEV-GFP BAC by KpnI digestion.

To construct an infectious clone of TGEV, six overlapping cDNA fragments designated A to F were generated by reverse transcriptase PCR (RT-PCR) using total RNA extracted from PK-15 cells infected with TGEV WH-1 (Figure 1A,B). Fragments A, B, D, and E were cloned into the pMD18-T vector, and fragments C and F were cloned into the bacterial artificial chromosome (BAC) to produce the corresponding subclones. Subclone F was also constructed as the final recipient BAC vector by inserting the cytomegalovirus (CMV) promoter at the 5′ terminus of fragment F and a 25-bp poly(A) tail (25A) followed by the hepatitis delta virus ribozyme (Rz) and bovine growth hormone (BGH) transcription terminal signal sequences at the 3′ terminus of fragment F (Figure 1B). To more conveniently observe the chimeric virus and exclude the influence of the accessory gene *ORF3* on TGEV enteric tropism and virulence, the gene encoding *ORF3* at the genome position from 24,826 to 28,580 was replaced by the *EGFP* gene (Figure 1A,B). Through the one-step assembly of fragments A to F, we successfully obtained a full-length cDNA infectious clone of TGEV, designated pTGEV-GFP BAC (Figure 1B,C). The full-length pTGEV-GFP BAC was verified by sequencing. After propagation for more than 200 generations in *E. coli* DH10B cells, pTGEV-GFP BAC digestion with the KpnI enzyme, which produced six different fragment products, also confirmed the correct pTGEV-GFP BAC clone (Figure 1C,D). In other words, the cloning of fragment C into the BAC yielded no toxic sequences in any of the experiments.

**Figure 2:**
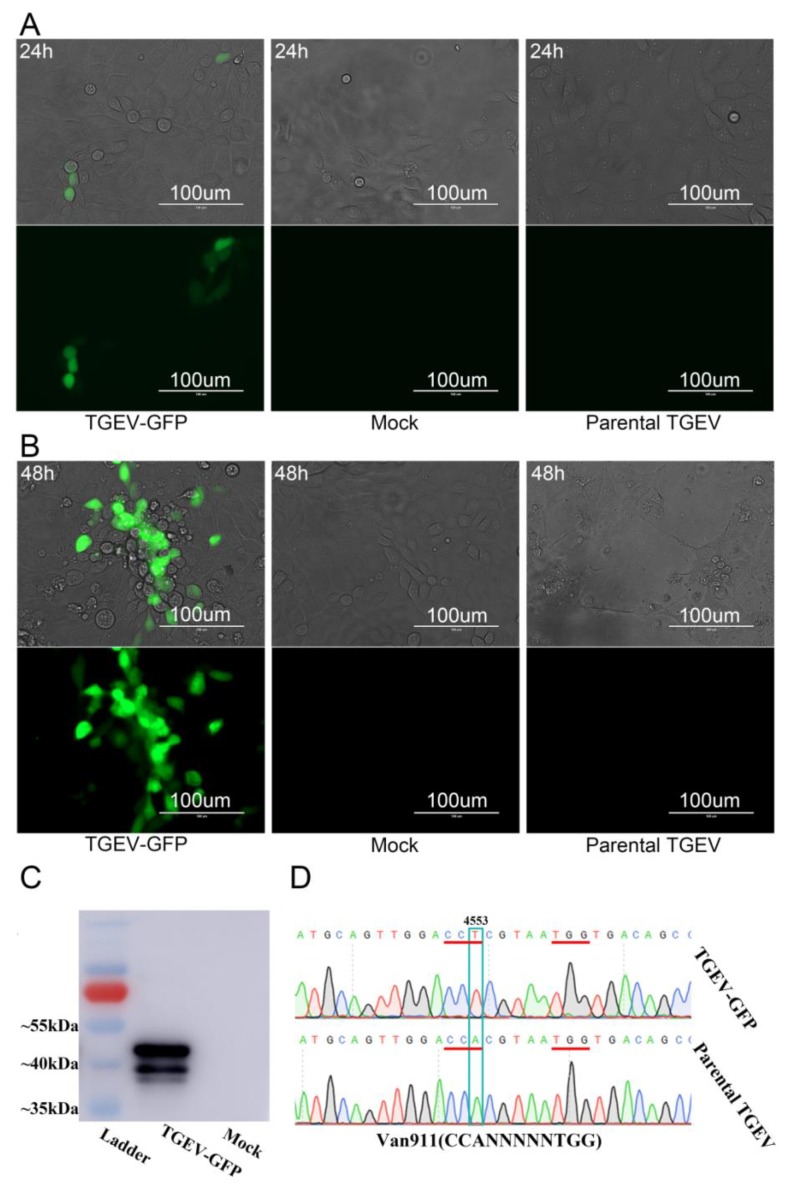
Rescue of the TGEV-GFP infectious clone in PK-15 cells. (**A**) PK-15 cells were infected with the recombinant or parental virus or mock infected, and at 24 h post-infection, their green fluorescence was visualized. (**B**) CPE or green fluorescence of PK-15 cells infected with the recombinant viruses TGEV-GFP or parental TGEV or mock infected was visualized at 48 h post-infection. (**C**) The expression of the TGEV N protein in PK-15 cells infected with recombinant TGEV-GFP or mock infected was analyzed by Western blotting using rabbit poly-antiserum against TGEV N protein. (**D**) The mutation of A4553T at nt 4553 was determined by sequencing after performing RT-PCR in triplicate.

To rescue the recombinant CoV of TGEV-GFP (corresponding to pTGEV-GFP BAC), the pTGEV-GFP BAC was transfected into PK-15 cells using Lipofectamine 2000. Sporadic green fluorescence was observed 24 h post-transfection, as depicted in Figure 2A, but the infected PK-15 cells grew and showed a normal morphology. However, compared with the mock-infected group, obvious green fluorescence and the cytopathic effect (CPE) could be observed 48 h post-transfection (Figure 2B). Western blot and RT-PCR assays were performed to further confirm the recombinant virus TGEV-GFP. The 43.5-kilodalton band of TGEV N protein (Figure 2C) and the marker mutation at position 4553 in the TGEV genome (Figure 1B and Figure 2D) confirmed the recovery of the TGEV-GFP recombinant virus.

### 3.2. Establishment of a Novel Approach for Coronavirus Gene Editing Using the CRISPR-Cas9 System

**Figure 3:**
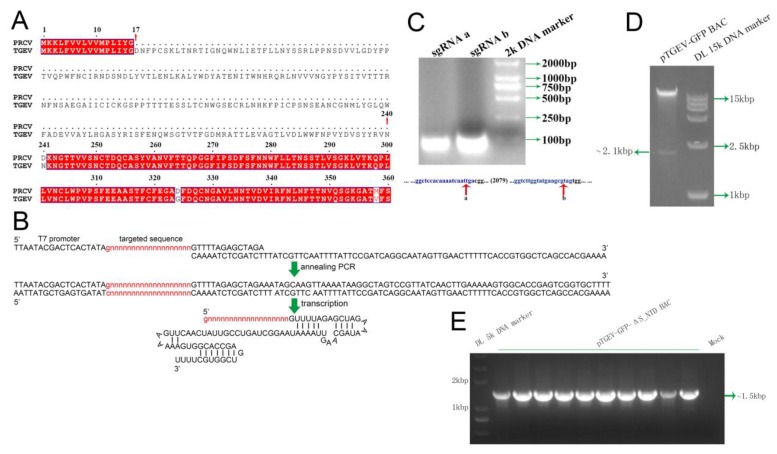
Specific cleavage of pTGEV-GFP BAC by the CRISPR/Cas9 system in vitro. (**A**) The specific sequence at residues 17-240 were deleted from the N-terminal domain of the TGEV *Spike* gene. The GenBank accession number of the PRCV S protein partial sequence is BAG83239.1. (**B**) Annealing PCR with the two specific primers was performed to transcribe the targeted sgRNA in vitro. (**C**) Electrophoresis detection of the purity of the transcription product from the annealing DNA fragments. The targeted sites a and b of pTGEV-GFP used for digestion are marked with red arrows. (**D**) Specific cleavage of pTGEV-GFP to delete the sequence containing S_NTD224. The pTGEV-GFP BAC digested by Cas9, guided by sgRNA a and sgRNA b, was detected by electrophoresis. (**E**) Electrophoresis identification of the pTGEV-GFP-ΔS_NTD by RT-PCR using a pair of primers (rec-672SF and rec-672SR).

Spike with N-terminal 224 aa deletion in TGEV WH1 is analogous to the spike of a reported natural PRCV strain (Figure 3A). To verify whether S_NTD224 is the enteric tropism determinant for TGEV, we used the CRISPR-Cas9 system to efficiently manipulate the TGEV gene. Briefly, two specific enzyme sites encompassing the sequence of S_NTD224 were selected. We then synthesized two types of single-stranded DNA forward primers (ssDNAa-F or ssDNAb-F) and a constant reverse primer (ssDNA-R) (Table 1) corresponding to the two enzyme cutting sites, designated sites a and b (Figure 3C). After annealing PCR using the forward and reverse primers, the purified PCR products of short DNA fragments were transcribed by T7 RNA polymerase (Figure 3B). The transcribed products corresponding to sites a and b (designated sgRNA a and sgRNA b) (Figure 3C) were incubated with the nuclease Cas9 to digest the pTGEV-GFP BAC in vitro, and the digestion yielded a linearized BAC and a ~2.1-kb DNA fragment that included the sequence of S_NTD224 (Figure 3A,D).

To construct the mutant infectious clone of the S_NTD224 deletion (designated pTGEV-GFP-ΔS_NTD BAC) from the pTGEV-GFP BAC, we produced a 672-nucleotide deletion-specific mutation PCR product using two pairs of primers (rec-672SF/δS-NTDR and rec-672SR/δS-NTDF) (Table 2). The mutation PCR products were then recombined into the linearized BAC vector cleaved from the full-length pTGEV-GFP BAC (Figure 3D). After the recombination products were transformed into DH10B competent cells, all 10 monoclonal colonies were identified as positive clones by PCR using the primer pair rec-672SF/rec-672SR (Figure 3E). The sequencing of three randomly selected monoclonal colonies also confirmed the positive pTGEV-GFP-ΔS_NTD BAC, and we then constructed an infectious clone with the corresponding 224-aa deletion in the N-terminus of the TGEV-GFP S protein by sequencing one entire genome of the three positive clone (Figure 3E).

### 3.3. Recovery and Characteristics of the Mutant Virus TGEV-GFP-ΔS_NTD in PK-15 Cells

**Figure 4:**
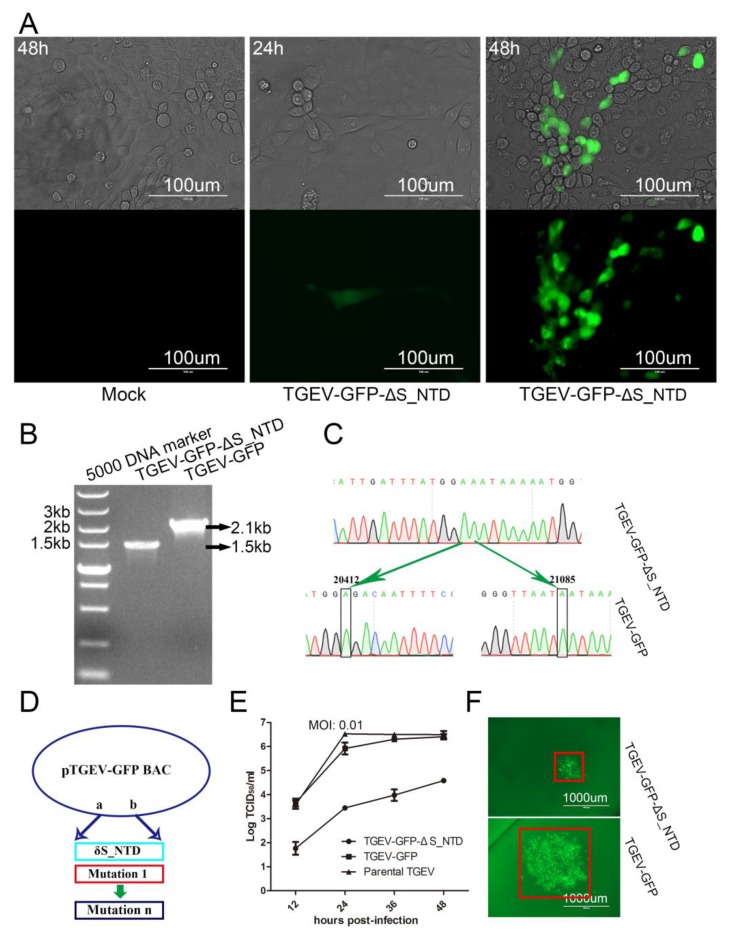
Rescue of TGEV-GFP-ΔS_NTD infectious clone in PK-15 cells. (**A**) CPE or fluorescence microscopy of PK-15 cells infected with the recombinant virus TGEV-GFP-ΔS_NTD or mock infected at 24 and 48 h post-transfection. (**B**) Electrophoresis detection of recombinant TGEV-GFP and TGEV-GFP-ΔS_NTD by RT-PCR using the primers rec-672SF and rec-672SR; (**C**) Sequence analysis of the targeted mutation area between recombinant TGEV-GFP and TGEV-GFP-ΔS_NTD by RT-PCR sequencing. (**D**) Model for the simultaneous construction of numerous infectious clones including S_NTD224. (**E**) Growth curves with the wild-type viruses TGEV-GFP and TGEV-GFP-ΔS_NTD with an original MOI of 0.01. (**F**) Viral fluorescent plaques between recombinant TGEV-GFP and TGEV-GFP-ΔS_NTD. The red box represents the size of the viral fluorescent plaques.

We rescued the recombinant virus TGEV-GFP-ΔS_NTD from PK-15 cells as previously described, and sporadic and more obvious green fluorescence was observed at 24 and 48 h post-transfection, respectively (Figure 4A). We then verified the modified virus TGEV-GFP-ΔS_NTD by RT-PCR using the primers PrimerF and PrimerR (Table 2). An obvious deletion of approximately 600 bp was observed in TGEV-GFP-ΔS_NTD in comparison with TGEV-GFP (Figure 4B). Subsequently, the RT-PCR product was sequenced using the primers PrimerF and PrimerR (Figure 4C). Comparison with the pTGEV-GFP BAC showed that all the modified nucleotides were correct, as depicted by the model shown in Figure 4D.

To further evaluate the role of S_NTD224 in TGEV, we measured the growth kinetics of the wild-type virus, TGEV-GFP and TGEV-GFP-ΔS_NTD. The replication kinetics of the TGEV-GFP and wild-type viruses were comparable to each other and considerably different from those of TGEV-GFP-ΔS_NTD (Figure 4E). Twelve hours after inoculation, the titer of TGEV-GFP was more than 10-fold greater than that of TGEV-GFP-ΔS_NTD (Figure 4E). To further identify the effect of S_NTD224 on TGEV, we also analyzed the fluorescent viral plaques. The plaque size of TGEV-GFP-ΔS_NTD was notably different from that of TGEV-GFP (Figure 4F), which also indicated that TGEV-GFP infected cells more effectively than TGEV-GFP-ΔS_NTD.

### 3.4. S_NTD224 Is Not the Enteric Tropism Determinant for TGEV

**Figure 5:**
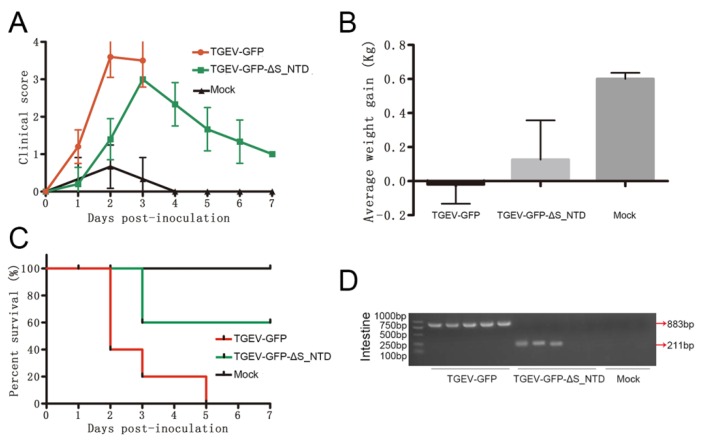
Pathogenicity evaluation and detection of the recombinant virus. (**A**) Clinical status scores of the piglets: 0, normal piglets; 1, piglets that moved slowly; 2, piglets that tended to lie down; 3, piglets that often lied down; and 4, piglets that were unstable to stand or moribund. (**B**) Average weight gain of the piglets at the time of euthanasia. (**C**) Percent survival of the different piglet groups. (**D**) Virus detection in intestinal tissue by nested-RT-PCR. All intestinal tissue samples were collected when the piglets were moribund or euthanized at 7 days post inoculation.

To validate whether S_NTD224 determines the enteric tropism of TGEV, 13 two-day-old piglets were randomly divided into three groups, with five piglets in each virus-infected group and three piglets in the mock-infected control group. The piglets in the two virus-infected groups were inoculated intranasally and orally at a dose of 1 × 10^5^ 50% tissue culture infective dose (TCID_50_) with the respective chimeric virus, and the mock-infected control piglets were inoculated with Dulbecco’s modified Eagle’s medium (DMEM). All the piglets in the TGEV-GFP group exhibited severe clinical symptoms and weight loss, and those in the TGEV-GFP-ΔS_NTD group showed ameliorated but still obvious clinical symptoms (Figure 5A,B). In addition, the piglets in the TGEV-GFP and TGEV-GFP-ΔS_NTD groups appeared moribund within 3 days postinoculation, whereas the piglets in the mock-infected control group remained healthy (Figure 5C). In addition, the piglets in the TGEV-GFP group showed a higher mortality rate (as high as 100%) and presented earlier symptoms compared with those in the TGEV-GFP-ΔS_NTD group, which showed 40% mortality at 3 days postinoculation (Figure 5A,C). To better detect the presence of the inoculated virus in the euthanized piglet intestine, the presence of both TGEV-GFP and TGEV-GFP-ΔS_NTD in intestinal tissue was detected by nested PCR using the primer pairs F1/R1 and F2/R2 (Table 2). TGEV-GFP and TGEV-GFP-ΔS_NTD were detected in intestinal tissue from the moribund piglets (Figure 5D), but no chimeric virus was detected in the two piglets in the TGEV-GFP-ΔS_NTD group that were euthanized at 7 days post-inoculation (Figure 5D).

**Figure 6:**
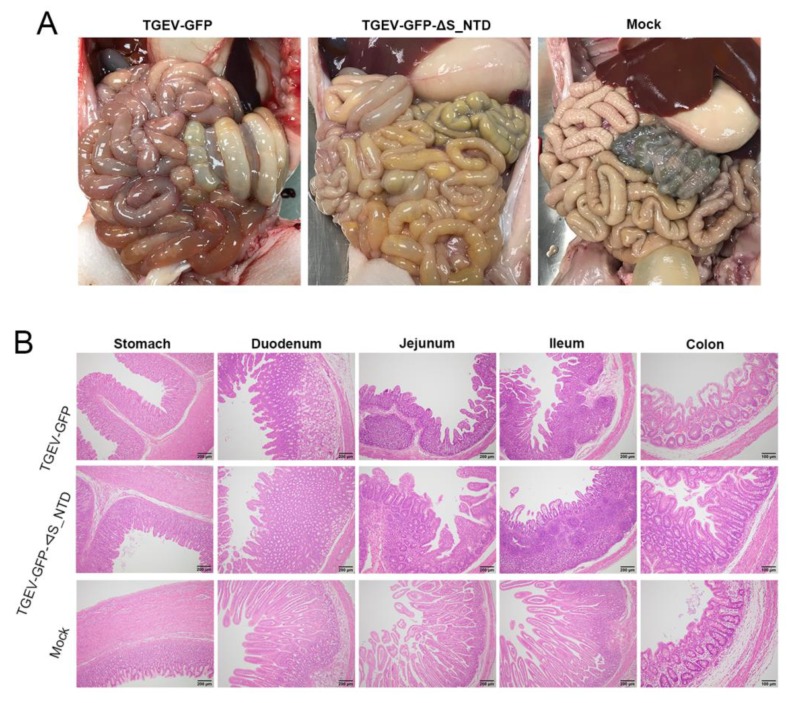
Gross lesions and histopathological examination of piglets challenged by recombinant viruses: (**A**) Gross lesions after the piglets were euthanized. The piglets in the TGEV-GFP and TGEV-GFP-ΔS_NTD groups showed obvious colon damage compared with those of the mock group. (**B**) Histopathological examination of different tissue sections, including the stomach, duodenum, jejunum, ileum and colon, from the piglets.

The postmortem of the moribund piglets in the TGEV-GFP and TGEV-GFP-ΔS_NTD groups revealed that the small intestines were filled with watery contents. In particular, the intestinal walls in the jejunal section of the intestines of these piglets were clearly thinner and more transparent compared with those of the mock group (Figure 6A). HE staining compared with the normal mock group also revealed that the TGEV-GFP chimeric viruses caused more severe intestinal tissue damage than TGEV-GFP-ΔS_NTD (Figure 6B). More severe villous atrophy was observed in the small intestine, particularly the jejunum and ileum, of the piglets in the TGEV-GFP and TGEV-GFP-ΔS_NTD groups compared with those of the mock group (Figure 6B). Collectively, these results suggested that S_NTD224 has not altered the enteric tropism for TGEV but exerts a mild influence on TGEV virulence.

## 4. Discussion

The N-terminal domain of Spike protein is recognized as the TGEV enteric tropism determinant in piglets, as demonstrated through comparisons of the sequences of natural TGEV isolates or those obtained after continuous passage in cell culture [13,51,52], but more direct evidence is still needed. In this study, based on a DNA-launched infectious clone, we used a novel CoV gene editing method to efficiently perform CoV targeted gene editing. Using reverse genetics, we found that S_NTD224 was not the enteric tropism determinant for TGEV. The relevant insights regarding the novel CoV targeted gene editing method and TGEV S_NTD224 are discussed below.

### 4.1. Efficient Targeted CoV Gene Editing

Because CoVs are the viruses with the largest RNA genomes, the construction of a CoV infectious clone is hampered by two main challenges: large full-length cDNA and toxic sequences in the bacterial clone [31,53]. Although the problem of CoV cDNA sequence instability has been overcome by several methods, the manipulation of the large full-length CoV genome remains a considerable challenge. Until now, the direct editing of the full-length cDNA of CoVs has not been reported. In this study, we constructed a TGEV-GFP infectious clone by ligating six fragments in one step [49]. The CRISPR/Cas9 system was then used to finish the construction of pTGEV-GFP-ΔS_NTD. To our knowledge, this study provides the first demonstration of the direct in vitro manipulation of full-length coronavirus cDNA. To edit specific CoV genes, targeted cleavage of the BAC was completed by Cas9 protein through a reaction mediated by two types of sgRNA transcribed together or separately (Figure 3B,D). sgRNA can be easily obtained by annealing PCR and transcription using an available kit. Moreover, regardless of the exonuclease trimming activities of Cas9 [45], in the experiment, we were able to insert the mutated fragments in the linearized BAC with 200-bp overlapping sequences through homologous recombination.

Notably, numerous mutation fragments can be inserted efficiently into the linearized BAC at the same time (Figure 4D), which is perfect for the construction of a viral mutant library [54,55,56]. Similar to traditional plasmid manipulation, we edited the targeted gene by recombination in vitro with overlapping PCR products (e.g., mutations, deletions, or insertions). Moreover, as little as 100 ng of linearized, digested BAC was sufficient to complete the recombination reaction. To determine the mutation of the targeted BAC, we only needed to amplify the fragments by bacterial PCR using a pair of primers in duplicate, and this assay can be used to sequence the site of recombination and the modified fragment area. Furthermore, almost any area of the targeted BAC can be simply cleaved by the CRISPR/Cas9 system with two types of specific sgRNA. Throughout the process, we accomplished recombinant virus recovery using only one plasmid of BAC in a single week (Figure 3). Namely, once an infectious clone was constructed, the recombinant coronaviruses was more efficiently and conveniently rescued in this study than in previous research, and the proposed approach thus greatly accelerates the gene editing speed of large RNA virus rescue. Moreover, the simple manipulation of a BAC vector and modification of the specific small region throughout the procedure would theoretically lower the mutation probability of the full-length CoV cDNA. Thus, this method is not only cost-effective but also reduces the probability of introducing additional mutations during the BAC modification procedure.

### 4.2. S_NTD224 of TGEV Had a Mild Influence on TGEV Virulence but Was Not the Enteric Tropism and Virulence Determinant

The *Spike* gene of TGEV has been shown to alter TGEV virulence or enteric tropism. However, recombinant TGEV with S protein N-terminal amino-acid deletion was constructed through targeted recombination and passaged several times, which might cause other locus mutations in addition to the S protein deletion [20]. In particular, an early study reported that a 224-residue deletion in PRCV corresponding to the N-terminal domain of the TGEV S protein, as depicted in Figure 3A, is likely responsible for the loss of replication observed in the enteric tract [21]. No other studies have provided direct evidence demonstrating that only the N-terminal region of the *Spike* gene changes the TGEV virulence or enteric tropism [57]. Here, we emphasize the importance of the N-terminus of the TGEV S protein for the enteric tropism of the virus. To that end, we constructed a recombinant virus with an S protein analogous to that of PRCV (Figure 3A), TGEV-GFP-ΔS_NTD, and this recombinant virus showed titers and fluorescent plaque sizes that greatly differed from those of TGEV-GFP (Figure 4E,F). These results indicated that the 224 amino acids of the N-terminal of the TGEV Spike protein are not essential for viral survival but important for viral replication or infection, which was analogous to the findings obtained for PEDV and other CoVs [58,59,60,61]. Our animal experiments revealed that TGEV-GFP-ΔS_NTD caused 40% mortality in piglets and obvious intestinal tissue damage, which indicates that S_NTD224 has a mild influence on virulence but does not alter the enteric tropism of TGEV [62].

Using reverse genetics, we confirmed that changes in S_NTD224 alone altered, albeit not completely, the virulence of TGEV. One reason explaining no detection of TGEV-GFP-ΔS_NTD in the two piglets euthanized at 7 days post-inoculation might be related to immunity of recovering piglets. The role of S_NTD224 might be analogous to that of the 197-amino-acid region in the N-terminus of the PEDV S gene when used as a viral virulence marker. Consistent with previous reports, our experiments also detected TGEV-GFP and TGEV-GFP-ΔS_NTD in the jejunal contents tissue by nested RT-PCR [63,64], which indicates that changes in S_NTD224 alone do not alter TGEV enteric tropism in vivo. And it is also possible that other genes in addition to the 224 amino acids of the N-terminal of the TGEV Spike protein might regulate changes in TGEV tissue tropism [20,58]. Additional research is needed to determine the detailed mechanism of TGEV enteric tropism in vivo.

## 5. Conclusions

In summary, using the reverse genetics method, we have provided direct evidence showing that the N-terminal domain of Spike protein is not the determinant of TGEV enteric tropism in piglets, although S_NTD224 exerts a mild influence on TGEV virulence. These results provide new insights into the development of a new attenuated vaccine against TGEV. Furthermore, the method developed in this study allows the efficient and rapid editing of the full-length CoV genome in vitro and can theoretically be applied to all viruses with large RNA genomes once the full-length cDNA is obtained.

## Figures and Tables

**Table 1:** Details of the oligonucleotide primers used to produce the specific sgRNA for the double digestion of targeted BAC.

Primer	Sequence
ssDNAa-F	TTAATACGACTCACTATA GGCTCCACAAAATCAATTGA GTTTTAGA GCTAGA
ssDNAb-F	TTAATACGACTCACTATA GGTCTTGGTATGAAGCGTAG GTTTTAGA GCTAGA
ssDNA-R	AAAAGCACCGACTCGGTGCCACTTTTTCAAGTTGATAA CGGACTAGCCTTATTTTAACTTGCTATTTCTAGCTCTAAAAC

**Table 2:** Details of the oligonucleotide primers used to construct and detect the transmissible gastroenteritis virus (TGEV) S_NTD224 for recombination.

Primer	Sequence
rec-672SF	GATGGCTCCACAAAATCAA
δS-NTDR	GTAGTACCATTTTTATTTCCATAAATCAATGGCATTACG
δS-NTDF	AATAAAAATGGTACTACCGTAG
rec-672SR	TGGGTTGACCATAACCAC
PrimerF	GACGCAGACTTCAGTGTTAC
PrimerR	TCAGAACGAATACAGTACAC
F1	AGGGTAAGTTGCTCATTAGAAATAATGG
R1	CTTCTTCAAAGCTAGGGACTG
F2	TTGTGGTTTTGGTCGTAATGCC
R2	GGCTGTTTGGTAACTAATTTACCA
